# Pulmonary Artery Pseudoaneurysm: A Rare Cause of Fatal Massive Hemoptysis

**DOI:** 10.1155/2018/8251967

**Published:** 2018-04-23

**Authors:** Himaja Koneru, Sreeja Biswas Roy, Monirul Islam, Hesham Abdelrazek, Debabrata Bandyopadhyay, Nikhil Madan, Pradnya D. Patil, Tanmay S. Panchabhai

**Affiliations:** ^1^Department of Thoracic Medicine, Geisinger Medical Center, Danville, PA, USA; ^2^Norton Thoracic Institute, St. Joseph's Hospital and Medical Center, Phoenix, AZ, USA; ^3^Taussig Cancer Institute, Department of Hematology and Oncology, Cleveland Clinic, Cleveland, OH, USA

## Abstract

Pulmonary artery pseudoaneurysm (PAPA), an uncommon complication of pyogenic bacterial and fungal infections and related septic emboli, is associated with high mortality. The pulmonary artery (PA) lacks an adventitial wall; therefore, repeated endovascular seeding of the PA with septic emboli creates saccular dilations that are more likely to rupture than systemic arterial aneurysms. The most common clinical presentation of PAPA is massive hemoptysis and resultant worsening hypoxemia. Computed tomography angiography is the preferred diagnostic modality for PAPA; typical imaging patterns include focal outpouchings of contrast adjacent to a branch of the PA following the same contrast density as the PA in all phases of the study. In mycotic PAPAs, multiple synchronous lesions are often seen in segmental and subsegmental PAs due to ongoing embolic phenomena. The recommended approach for a mycotic PAPA is prolonged antimicrobial therapy; for massive hemoptysis, endovascular treatment (e.g., coil embolization, stenting, or embolization of the feeding vessel) is preferred. PAPA resection and lobectomy are a last resort, generally reserved for patients with uncontrolled hemoptysis or pleural hemorrhage. We present a case of a 28-year-old woman with necrotizing pneumonia from intravenous drug use who ultimately died from massive hemoptysis and shock after a ruptured PAPA.

## 1. Introduction

Pulmonary artery pseudoaneurysms (PAPAs) are associated with high mortality; therefore, early detection is of paramount importance. Massive hemoptysis from a ruptured PAPA is fatal in over 50% of patients, and a high index of suspicion is required for the diagnosis. These aneurysms frequently occur in patients with necrotizing infections of the lung or heart and those who are at high risk of septic embolism. We present the case of a 28-year-old woman who was admitted to our institution with prolonged respiratory failure. She was an intravenous drug user, and she had necrotizing pneumonia that led to development of multiple PAPAs, which in turn led to massive hemoptysis, shock, and death.

## 2. Case Report

A 28-year-old woman with a recent history of intravenous drug use presented with insomnia, generalized pain, and anxiety. Her physical examination was significant for tachycardia (heart rate: 130 beats per minute), hypoxia with oxygen saturation of 82% on room air, bilateral diffuse crackles over both lung fields, and confusion. Her initial laboratory evaluation revealed acute kidney damage (creatinine: 5.8 mg/dL). A chest radiograph demonstrated bilateral patchy alveolar opacities, indicating an acute alveolar process such as pulmonary edema or pneumonia. Ensuing hypoxic respiratory failure required endotracheal intubation. Given the patient's history of intravenous drug use, an emergent transesophageal echocardiogram was performed. This showed a large, bulky vegetation on the tricuspid valve that appeared to involve more than one leaflet, as well as possible pulmonic valve endocarditis with a small mobile echogenicity attached to the pulmonic valve, consistent with infective endocarditis.

Broad antimicrobial therapy was initiated with vancomycin and piperacillin-tazobactam. Blood cultures grew* Staphylococcus aureus*, as did the tracheal aspirate. Computed tomography (CT) of the chest, abdomen, and pelvis revealed numerous pulmonary septic emboli with hepatosplenomegaly. On day 4, antimicrobial coverage was changed to a daptomycin-based regimen on the sensitivities, but the patient's fever and tachycardia persisted.

Repeat CT of the chest, abdomen, and pelvis revealed interval worsening pulmonary opacities, new cavitary lesions, new bilateral lower-lobe-predominant consolidations, and a complicated right pleural effusion. Right thoracoscopy was performed and an intercostal drain was placed for the right empyema. Flexible fiber-optic bronchoscopy revealed copious purulent material in both lungs. Anticipating prolonged weaning from mechanical ventilation, a tracheostomy was performed on day 10. Repeat blood cultures on days 7, 9, and 20 showed no signs of bacterial infection, and the patient's overall clinical condition improved. She was weaned off mechanical ventilation on day 20.

On days 25 through 27, the patient began experiencing intermittent, self-limited episodes of hemoptysis via her tracheostomy tube. Fiber-optic bronchoscopy revealed small blood clots, but overall her clinical condition was improving. On day 34, the patient developed massive hemoptysis, and copious amounts of blood were suctioned from her mouth, nostrils, and tracheostomy tube. She rapidly developed progressive hypoxemia that was unresponsive to 100% supplemental oxygen; mechanical ventilation was therefore initiated. Hypoxic cardiorespiratory arrest ensued and after several rounds of cardiopulmonary resuscitation, return of spontaneous circulation was achieved.

**Figure 1:**
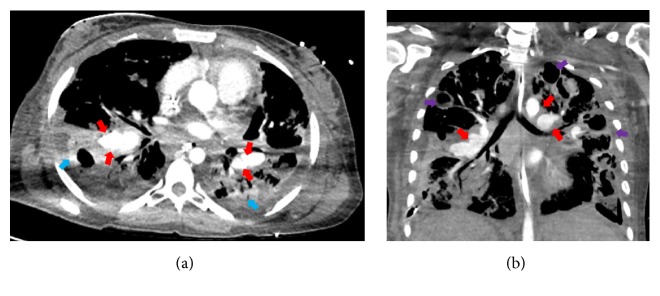
Computed tomogram angiography of the chest in (a) axial plane and (b) coronal plane depicting multiple saccular, contrast-enhanced structures close to the branches of the pulmonary arteries. These were found to be mycotic pulmonary artery pseudoaneurysms (red arrows). Bilateral, bibasilar dense consolidations from necrotizing* Staphylococcus aureus* pneumonia (blue arrows) and cavities secondary to necrotizing* S. aureus* pneumonia (purple arrows) are also seen.

Another fiber-optic bronchoscopy demonstrated massive blood clots throughout the patient's airways bilaterally, but no active source of bleeding was identified. She developed refractory shock that required multiple vasoactive agents, as well as blood products delivered via massive transfusion protocol. An emergent CT angiogram of the chest revealed bilateral PAPAs and a dilated right heart, likely related to pulmonary hypertension (Figure 1). After multidisciplinary review of her case with interventional radiology experts and thoracic surgeons, embolization was deemed inappropriate due to the size of the PAPAs. Bilateral lower lobe resection was considered, but it was thought to be futile given her unstable hemodynamics and underlying poor lung reserve from the ongoing* S. aureus* pneumonia. Moreover, such a procedure involved a high risk of massive hemorrhage of the large PAPAs. The patient's grim prognosis was discussed with her family, and she was transitioned to comfort care. She died on day 35.

## 3. Discussion

Although they are rare, the mortality of PAPAs is as high as 50% in diagnosed cases [1, 2]. PAPAs may be congenital or acquired, and the most common cause of acquired PAPA is infection [3]. Tuberculosis and syphilis were the former main infectious causes of PAPA; however, in the current era of efficacious antibiotic therapy, the incidence of PAPA due to tuberculosis or syphilis has greatly decreased [3]. Organisms implicated in causing modern-day PAPA include pyogenic bacteria (e.g.,* S. aureus*,* S. pyogenes*,* Klebsiella*, and* Actinomyces*),* Mycobacterium tuberculosis* (rare), and various fungi (*Mucor*,* Aspergillus*, and* Candida*) [4]. Successive endovascular seeding of the PA lumen from multiple emboli or microemboli has been proposed as the pathogenesis of PAPAs in the infectious setting [5]. Such repeated seeding gradually destroys the vessel wall from the inside (i.e., the luminal side) outward, causing PAPA formation. Other rare causes of PAPAs include chest wall trauma and iatrogenic trauma from PA catheters. Several case reports have described PAPAs resulting from lung cancer (including primary squamous cell cancer, primary sarcoma, and metastatic sarcoma) [4, 6–8].

Patients with PAPA commonly present with hemoptysis and hypoxemia and sometimes experience chest pain. Chest radiography can show focal consolidation, solitary pulmonary nodules, or multiple pulmonary nodules near the central or peripheral pulmonary vasculature [9]. Definitive diagnosis is made via CT angiography—a modality that not only establishes diagnosis, but also helps plan therapy with endovascular treatment modalities. On CT angiography, PAPAs appear as focal outpouchings of contrast adjacent to a PA branch following the same contrast density as the PA in all phases of the study [10]. Pulmonary angiography demonstrates delayed emptying of contrast material from the sac [10]. In the case of a mycotic PAPA, multiple synchronous lesions are usually seen in segmental and subsegmental PAs due to ongoing embolic phenomena.

Because the PA lacks an adventitial wall, PAPAs are more likely to rupture than true arterial aneurysms. Therefore, hemoptysis due to a ruptured PAPA is often fatal and must be promptly recognized and treated. Antimicrobial therapy remains an important component of managing mycotic PAPAs. Empiric intravenous antimicrobial therapy targeting broad gram-negative and gram-positive coverage should be instituted as soon as PAPA is suspected. In addition, antifungal, antimycobacterial, and antitreponemal coverage must be considered in immunocompromised patients. Although no overall consensus exists regarding the optimal duration and type of antimicrobial therapy in patients with PAPA, prolonged therapy similar to infective endocarditis is considered appropriate.

Urgent endovascular treatment is the preferred approach in managing hemoptysis resulting from PAPAs, and such treatment should not be delayed. Direct coil embolization, endovascular stents, or embolization of the aneurysm's feeding vessel are the reported effective occlusion modalities [11–14]. In the case of a small pseudoaneurysm, occluding the feeding vessel might be adequate; however, coil embolization is preferred for larger aneurysms [11–14]. It is important to note that successful embolization of the PAPA not only requires successful coil placement, but also an intact coagulation cascade and reduced arterial pressure to promote thrombosis. This is a challenge, especially in mycotic PAPAs, as patients usually present with sepsis and associated coagulopathy. Balloon embolization can be considered as an alternative in these cases [15]. In wide-necked PAPAs, endovascular stenting has been described as successful treatment [16, 17]. Potential graft infection due to ongoing septic embolic phenomena must be considered as a possible complication of graft stent placements and this treatment should be avoided in patients with active bacteremia, due to the high chances of graft seeding. Another less preferred approach in the management of PAPAs that are not amenable to endovascular treatment is direct percutaneous thrombin injection [18].

Operative repair for PAPAs involves open thoracotomy and aneurysm resection, with lobectomy for the involved lobes. Surgical treatment, however, is associated with increased mortality and morbidity compared with endovascular treatment, especially because patients with mycotic PAPAs are acutely ill and often have poor pulmonary reserve [19, 20]. Surgical approaches should be reserved for patients with pleural hemorrhage, uncontrolled hemoptysis, or aggressive infections that may not respond to medical therapy alone (e.g., mucormycosis). Surgery is also preferred in patients who have a high chance of endovascular graft or coil infection, such as patients with active bacteremia or resistant organisms [19, 20]. In conclusion, PAPAs are rare complications of infective endocarditis. A high degree of suspicion is needed for diagnosing PAPAs on imaging. Timely recognition can improve outcomes, as it allows for earlier intervention.
